# Correlation between investment in sexual traits and valve sexual dimorphism in *Cyprideis* species (Ostracoda)

**DOI:** 10.1371/journal.pone.0177791

**Published:** 2017-07-05

**Authors:** Maria João Fernandes Martins, Gene Hunt, Rowan Lockwood, John P. Swaddle, David J. Horne

**Affiliations:** 1 Department of Paleobiology, National Museum of Natural History, Smithsonian Institution, Washington DC, United States of America; 2 Department of Geology, College of William and Mary, Williamsburg, Virginia, United States of America; 3 Department of Biology, College of William and Mary, Williamsburg, Virginia, United States of America; 4 School of Geography, Queen Mary University of London, London, United Kingdom; Seconda Universita degli Studi di Napoli, ITALY

## Abstract

Assessing the long-term macroevolutionary consequences of sexual selection has been hampered by the difficulty of studying this process in the fossil record. Cytheroid ostracodes offer an excellent system to explore sexual selection in the fossil record because their readily fossilized carapaces are sexually dimorphic. Specifically, males are relatively more elongate than females in this superfamily. This sexual shape difference is thought to arise so that males carapaces can accommodate their very large copulatory apparatus, which can account for up to one-third of body volume. Here we test this widely held explanation for sexual dimorphism in cytheroid ostracodes by correlating investment in male genitalia, a trait in which sexual selection is seen as the main evolutionary driver, with sexual dimorphism of carapace in the genus *Cyprideis*. We analyzed specimens collected in the field (*C*. *salebrosa*, USA; *C*. *torosa*, UK) and from collections of the National Museum of Natural History, Washington, DC (*C*. *mexicana*). We digitized valve outlines in lateral view to obtain measures of size (valve area) and shape (elongation, measured as length to height ratio), and obtained several dimensions from two components of the hemipenis: the muscular basal capsule, which functions as a sperm pump, and the section that includes the intromittent organ (terminal extension). In addition to the assessment of this primary sexual trait, we also quantified two dimensions of the male secondary sexual trait—where the transformed right walking leg functions as a clasping organ during mating. We also measured linear dimensions from four limbs as indicators of overall (soft-part) body size, and assessed allometry of the soft anatomy. We observed significant correlations in males between valve size, but not elongation, and distinct structural parts of the hemipenis, even after accounting for their shared correlation with overall body size. We also found weak but significant positive correlation between valve elongation and the degree of sexual dimorphism of the walking leg, but only in *C*. *torosa*. The correlation between the hemipenis parts, especially basal capsule size and male valve size dimorphism suggests that sexual selection on sperm size, quantity, and/or efficiency of transfer may drive sexual size dimorphism in these species, although we cannot exclude other aspects of sexual and natural selection.

## Introduction

Although sexual dimorphism is commonly thought to evolve via sexual selection [1], the relationships between the strength and direction of selection and the occurrence of sexual dimorphism have seldom been quantified [2–4]. The assumption that sexual dimorphism is the product of sexual selection has led many researchers to use the extent of dimorphism between males and females as a proxy for the strength of sexual selection (see review by [5]). However, a few studies have indicated that dimorphism in size, color, and even behavioral traits can result from evolutionary change in females, and not as a direct product of sexual selection [3, 4, 6]. Therefore, we should not assume that sexual dimorphism is the result of sexual selection without investigating the cause of the difference between males and females. If it is possible to use dimorphism as an indicator of sexual selection, then it would be possible to test comparative questions related to the ultimate outcomes of sexual selection—such as those that predict sexual selection could lead to differential rates of speciation and extinction [7–13].

One way of investigating the cause of sexual dimorphism is to examine whether variation in primary, as well as secondary, sexual traits is associated with dimorphism. If sexual dimorphism positively covaries with primary sexual traits, such as gonads and genitalia, then it seems likely that the dimorphism results from increased investment in sexual processes, and would be consistent with the interpretation of dimorphism resulting from sexual selection [7, 14, 15]. Here, we focused on the covariation of male genitalia and carapace size and shape of ostracodes—small, bivalved crustaceans. We chose to study male genitalia as these traits often evolve rapidly [14, 15] and are often key traits in distinguishing close relatives (e.g., insects: [14]; ostracodes: [16, 17]). Most accepted hypotheses for the rapid evolution of genital morphology and secondary sexual traits fit under the umbrella of sexual selection, although the specific mechanisms sometimes remain unclear [7, 14, 15].

The Ostracoda are characterized by the evolution of a highly diverse copulatory apparatus, the hemipenis, which includes a muscular sperm pump and an intromittent organ and associated features. These structured are bilaterally paired and very large; together they can occupy up to a third of the carapace volume [16]. A sperm pump is needed to transfer the sperm, a non-trivial task given that ostracodes are known for having long sperm (30–10,000 μm; [18–20]); exceptionally preserved fossils indicate that this trait has persisted since at least the Cretaceous (e.g., [20, 21]). It appears highly likely that the large penis, sperm pump, and large sperm all evolved via sexual selection [16]. Investment in male genitalia is indicative of more intense sperm competition [22], cryptic female choice [23], and sexual conflict [24, 25] in other taxa.

In ostracodes, investment in genitalia is thought to have consequences for the size and shape of their valves [16, 26, 27]. In the superfamily Cytheroidea, male valves are relatively more elongate than female valves, especially in the posterior region, a style of dimorphism that traditionally has been explained in terms of the male valves accommodating the large hemipenis. This explanation is logical, but to our knowledge, has never been thoroughly tested. Here we test this widely held prediction that male investment in primary sexual structures, and potentially also secondary sexual structures, correlates directly with sexual dimorphism of the shell in three species of the ostracode genus *Cyprideis*. Because of the large size of the muscular pump relative to the rest of the hemipenis, we also predict that correlations with valve dimensions will be strongest for this structure.

## Material & methods

### Ethics statement

The present study complied with federal and state laws as the three *Cyprideis* species are not considered endangered nor have a protected species status. Sampling was carried out on public lands with no permissions required, or in the case of the UK sample with the permission of the Sandwich and Pegwell Bay National Nature Reserve.

### Study organism

Sexual dimorphism is strong but variable within the genus *Cyprideis* [28], with males relatively more elongate than females as in other cytheroideans [28]. The genus is represented by ca. 40 extant species [29], with over 70 fossil taxa recognized across Eurasia and the Americas. Its members are considered brackish-water inhabitants, although they can be found in habitats that range from freshwater to fully marine. The type species of the genus, *Cyprideis torosa*, has been reported in all salinities [17, 28]. A video of the *C*. *torosa* is provided as S1 File.

### Sample localities for *Cyprideis mexicana*, *C*. *salebrosa* and *C*. *torosa*

Specimens were opportunistically collected in the field or obtained from museum collections at the National Museum of Natural History (NMNH), Smithsonian Institution, Washington DC. *Cyprideis salebrosa* van den Bold (SALE) individuals were collected from several locations in Chesapeake Bay, Maryland, USA near the Smithsonian Environmental Research Center (SERC) field station; three samples were pooled for analysis (S1 Table). Specimens of *C*. *torosa* (Sharpe) (TORO) were collected July 2015 in Pegwell Bay, Kent, UK (S1 Table). Field samples were washed, sorted using a stereomicroscope, and kept in ~70% ethanol until dissection. *Cyprideis mexicana* Sandberg (MEXI) specimens were drawn from the NMNH collections. They were collected over 50 years ago by Louis S. Kornicker (NMNH) in Copano and Redfish Bays, Laguna Madre, Texas, USA (S1 Table). The samples designated as *Cyprideis* sp. by L. S. Kornicker and C. E. King (USNM 128367) contained several males of *C*. *mexicana*, in addition to males assignable to *C*. *gelica* and mixed juveniles. The specimens assigned to the *Cyprideis bensoni* lot by the same authors, with the catalog number USNM 128369, were composed of females of *C*. *mexicana* and females of a different, though similar, species. The sample USNM 128389, also part of the *Cyprideis bensoni* lot, was composed of juveniles and five males of *C*. *mexicana*.

Identifications to the species level followed [28, 30], and were confirmed by reference to holotype preparations of *C*. *mexicana* and *C*. *salebrosa* from the NMNH invertebrate zoology collection. The soft parts of different species of *Cyprideis* can be very similar, and we found the hemipenis to be the most reliable character in species identification [30]. Only fully mature adults were used in the analysis. Several adult males had incompletely chitinized hemipenis, probably representing very recently molted adults, and these were excluded from the analysis (see [31] for a similar phenomenon in cypridoid ostracodes). Terminology for soft anatomy followed [17] except for the hemipenis, for which we generally followed [17, 30, 32].

Final preparations for species identification and soft part imaging were set in glass slides with ostracodes dissected in 100% glycerin using entomologic needles. Shells were stored in cardboard micropaleontological slides. Dissections were carried using an Olympus SZX12 microscope. Soft parts and valves were imaged with an Olympus QColour5 digital camera using Q-Capture Pro 7 image analysis software (QImaging). Valves were photographed at 22.5x magnification; soft parts were photographed using transmitted light at 160x (hemipenis) and 224x (remaining limbs). Additional, z-stacked images taken for presentation purposes using an Olympus BX63 microscope and Olympus DP80 camera, using the Cellsens Dimension v. 1.13 image analysis software. Final preparations are stored at the NMNH in the Department of Invertebrate Zoology (S1 Table).

### Morphometric processing

**Fig 1:**
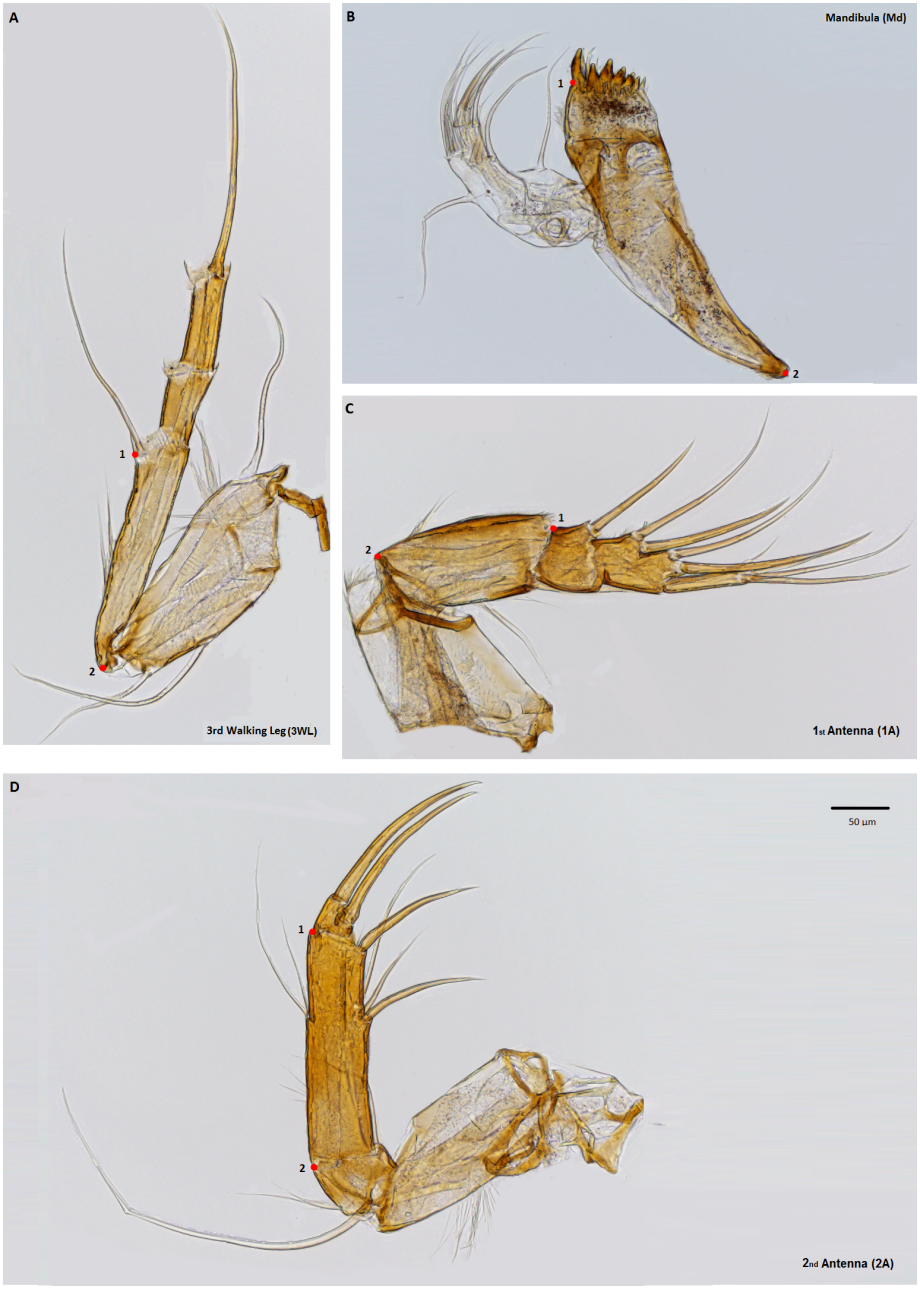
Illustration with landmark positions used to characterize length of the four reference limbs used to characterize soft-part body size, here represented by *C*. *salebrosa*. A) 3^rd^ walking leg, B) mandibular, C) 1^st^ antenna, D) 2^nd^ antenna. Abbreviations follow Table 1.

**Fig 2:**
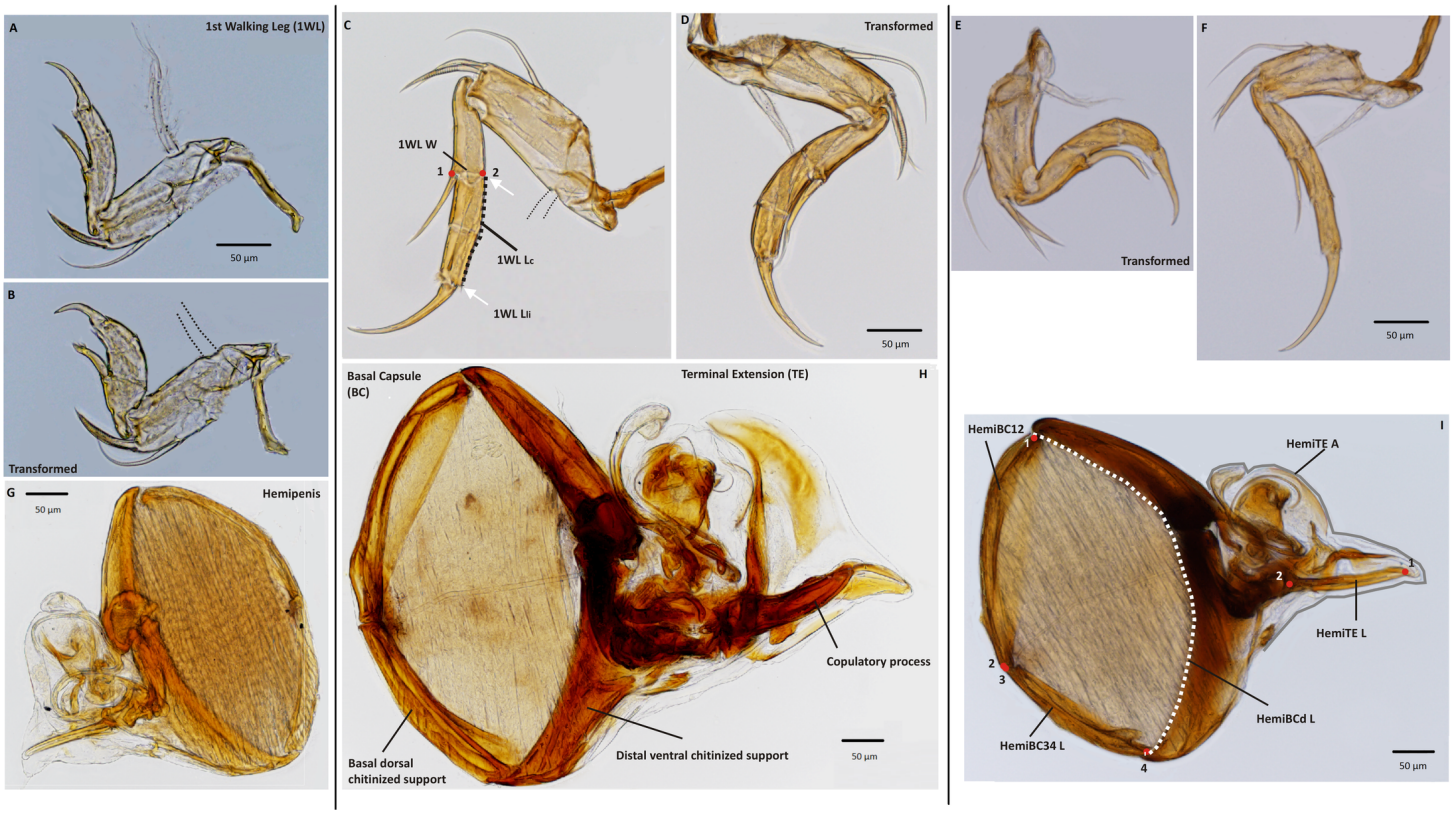
Illustration with landmark positions (red dots and white arrow for linear length) and semi-landmarks (dotted lines for curve length and grey solid line for area) on male *Cyprideis* sexual structures. A–F) 1^st^ walking leg (1WL), where the right side is indicated as transformed. C) Landmark positions and abbreviations as used in Table 1. G–I) Hemipenis. H) Indication of both units of the *Cyprideis* hemipenis, with terminology for specific components following Table 1. I) Landmark positions and abbreviations as used in Table 1. Left panel–*C*. *mexicana*. Middle panel–*C*. *salebrosa*. Right panel–*C*. *torosa*. Structures lost during specimen preparation indicated with grey dotted lines.

Anatomical landmarks and semi-landmarks along curves were used to quantify the length and shape of structures, and outlines were used to quantify area. Digitization was done with the software TpsDig 2.14 [33] and custom R-scripts were used to extract the data from TPS files. A detailed description of landmark definitions and abbreviations is given in Table 1; positions of landmarks are illustrated for each limb in Figs 1 and 2, with terminology and abbreviations following Table 1.

**Table 1:** Abbreviation and nomenclature applied in the morphological analysis. Descriptions of landmarks and semi-landmarks used to obtain linear dimensions, curve lengths, and areas of appendages.

Character	Abbreviation	Description	Data /Illustration
*Shell*			Table 2
Area	Size	Area of the valve outline.	Table 2
Length/Height	Shape	Length and height were estimated as the major and minor semi-axes of an ellipse fit to the valve outline.	Table 2
*Reference limbs*			Fig 1
First antenna	1A	Length of the I segment of the podomere, with the landmarks placed on the anterior margin at the junction with the II podomere (1), and the protopodite (2).	Table 3; Fig 1C
Second antenna	2A	Length of the II segment of the podomere, with the landmarks placed on the outer margin at the junction with the III podomere (1), and the I podomere (2).	Table 3; Fig 1D
Mandibula	Md	Landmark (1) positioned at the base of the teeth on the margin bearing the setose palp and the (2) landmark positioned at the terminal tip.	Table 3; Fig 1B
Third walking leg	3WL	Length of the I segment of the podomere, with landmark (1) positioned on the anterior margin at the base of the 'e' setae, and landmark (2) at the juntion on the proximal corner.	Table 3; Fig 1A
*Primary sexual character*			Fig 2G–2I
Basal Capsule	BC	Sperm pump unit, bounded on all sides by four chitinous supports.	Fig 2H
Basal Capsule distal chitinous support length	HemiBCd L	Curve length of the distal chitinized support. Semi-landmarks position along the inner margin of the bars (vesicle ejaculatory margin extending inwards over the muscular section was not included).	Table 4; Fig 2I
Basal Capsule basal dorsal chitinous support length	HemiBC12 L	Linear length of the basal ventral bar with landmarks (1–2) at the extremities.	S2 Table; Fig 2I
Basal Capsule basal ventral chitinous support length	HemiBC34 L	Linear length of the basal dorsal bar with landmarks (3–4) at the extremities.	S2 Table; Fig 2I
Basal Capsule size	HemiBC	Average obtained for the three lengths recovered on the BC.	
Terminal Extension	TE	Roughly triangular shaped unit composed of the copulatory complex and the labyrinth (including copulatory pipe), and including distally the intromittent organ (copulatory process).	Fig 2H
Copulatory process length	HemiTE L	Length of the intromittent organ (copulatory process).	Table 4; Fig 2I
Terminal Extension area	HemiTE A	Area of the TE, obtained by outlining the soft tissue margin	S2 Table; Fig 2I
*Secondary sexual character*		The first thoracopod is a walking leg in the female cytheroids while in the male, the right side of the endopod is transformed and used as a clasping organ during copulation. Bilateral asymmetry observed in male and its use during mating gives the limb a secondary sexual character status.	Fig 2A–2F
First walking leg curve length	1WL L_c_	Curve length of the II and III segment of the podomere, with semi-landmarks positioned on the outer margin (opposite the 'e' setae).	Fig 2C
First walking leg linear length	1WL L_LI_	To obtain the degree of curvature the 1WL, curve length was compared to a linear distance that would be obtained if the podomere were not curved.	Fig 2C
First walking leg width	1WL W	Width of the II segment of the podomere, with landmarks positioned on the junction with the I segment, with landmark (1) positioned at the basis of the 'e' setae.	Fig 2C

### Valve measurements and sexual dimorphism

Valve size was measured as the area of the digitized outline in lateral view, log-transformed as is typically done in studies of growth and allometry [34, 35]. Valve shape was computed as the ratio of valve length to height, also log-transformed; lengths and heights were calculated as the major and minor axes of an ellipse fit to the valve outline. The magnitude of sexual dimorphism was computed as the difference in mean values between the sexes, M—F. The use of a log scale renders these differences proportional, and thus comparable, across species.

### Primary sexual character and biometric analysis

We measured dimensions of two distinct morphological units of the cytheroid penis: the Basal Capsule (BC; Table 1, Fig 2H and 2I), the muscular section that functions as a sperm pump, and the Terminal Extension (TE; Table 1, Fig 2H and 2I), the distal, roughly triangular part of the copulatory organ that includes the copulatory complex and copulatory process. For the basal capsule, we measured three lengths (Fig 2I) that we log-transformed and summed to produce an overall measure of BC size (Table 1). On the terminal extension, we measured the length of the copulatory process, which is the actual intromittent organ, as well as the area of the whole copulatory complex (Fig 2I).

### Secondary sexual character: 1^st^ walking leg

In *Cyprideis*, the right 1^st^ walking leg (1WL) is a secondary sexual structure used by the male to contact/clasp the females during mating [16, 17]. In males, this limb is broader and markedly curved compared with the left 1WL (Fig 2A–2F), which retains the shape observed in females. We measured the strength of this directional asymmetry in males as the difference between right and left (R—L) in width (1WL W, Fig 2C; Table 1) and degree of curvature. The latter (1WL L; Table 1) was measured as (L_*C*_*—*L_Li_)–(R_*C*_*—*R_Li_), in which *R* and *L* are right and left side, with the subscript *C* representing the length along the curve of the limb and the subscript *Li* as representing the shortest length between the start and end point (indicated as a dash curve and with white arrows, respectively, in Fig 2C). Lower values of this index indicate right walking legs that are more highly curved, relative to those on the left.

### Reference limbs to measure overall body size

To obtain a measure of body size related to softparts, linear dimensions were measured from four non-sexually dimorphic limbs: 1^st^ antenna (= antennula), 2^nd^ antenna, mandibula, and 3^rd^ thoracic or walking leg (1A, 2A, Md and 3WL respectively; see Table 1 for landmark positioning, as illustrated in Fig 1). These dimensions were log-transformed and then averaged to compute an overall measure of body size from soft parts.

Static allometries were computed for all soft anatomy variables separately within each species. Each softpart measure was regressed against valve area as the measure of body size. Valve area was square-root transformed first so as to maintain the same dimensionality between variables and thus the expectation of unit slope under isometry, except when regressed against the area of the terminal extension of the hemipenis (HemiTEA), which is of the same dimensionality as valve area.

### Combining data from left and right sides and imputing missing data

We measured all variables on left and right sides of the body whenever possible, though in some cases poor preservation or damage during dissection resulted in data from just the left or right side of the organism. In perfectly bilaterally symmetric organisms, one can freely substitute left and right values. However, valves in *Cyprideis* are not symmetric: left valves overlap the right valves and are larger and less elongate. We also detected subtle directional asymmetry in some of the limb variables, especially the mandible (see Results). We used the following procedure to combine data from left and right sides in a way that accounts for directional asymmetry. If only left data were available, these were used as is. If only right data were available, these were converted to left side equivalents using regressions separately for each species that predict left values given right values (*R*^2^ for left on right regressions ranged from 0.95 to 0.98). If both left and right data were available, they were combined as the average of the observed left side data and the left side value that was predicted by the right side data. In the absence of directional symmetry, the left-right regression has a slope of one and an intercept of zero, and this approach becomes equivalent to simply averaging left and right sides. In the course of some dissections, it became difficult to track which limbs were from which side of the body. Such data were omitted when assessing the left-right asymmetry, but they were included in other analyses (with the larger limb assigned to be the left) because left-right asymmetries were found to be small compared to differences between individuals.

Two variables described above represent composite variables from several measurements: softpart size and Basal capsule size (Table 1). Both of these matrices had a small amount of missing data: 2.7% for softpart size and 1.7% for HemiBC size. Rather than omitting specimens with just one or two missing values, we imputed missing data using the R package *Amelia* [36], which models observations as drawn from a multivariate normal distribution, an assumption that is consistent with the original data set according to Royston’s test of multivariate normality (*p*-values range from 0.09 to 0.792 in the three species for both datasets). Imputation was performed within each data set. For example, only basal capsule size variables use to impute other BC variables, and then only within species. Because the measurements that went into these composite variables were highly correlated (within-species pairwise correlations ranged from 0.42 to 0.73 for softpart size and 0.44 to 0.96 for HemiBC size) it is reasonable to impute missing data using the information from the remaining variables. The *Amelia* package generates stochastic complete datasets, 500 of which were averaged to obtain estimates of each missing value.

### Correlation between soft anatomy and valve sexual dimorphism

We tested if aspects of the soft anatomy account for sexual dimorphism in the size and shape of male valves. Three aspects of male sexual anatomy are of interest: the basal capsule of the hemipenis (composite variable HemiBC), the terminal extension of the hemipenis (represented by the Terminal extension area—HemiTE A, and the Copulatory process length—HemiTE L) and the directional asymmetry of the first walking leg (represented by the two measurements: WL1 W, WL1 L). Correlations of these traits with valve size and shape might be conflated by a shared correlation with overall body size. Therefore, we computed partial correlations between the male features and the shell features such that their shared correlation with softpart size was partialled out. This procedure is equivalent to regressing a male feature on softpart size, and also a shell feature on softpart size, and then computing the correlation coefficient between both sets of residuals. This yields the same *p*-values as those on the relevant multiple regression (shell part ~ softpart size + male part); partial correlations can be thought of as regression coefficients that are scaled so that the strength of the relationship is more easily interpreted.

## Results

### Variation between sexes, between left and right sides, and among individuals

**Fig 3:**
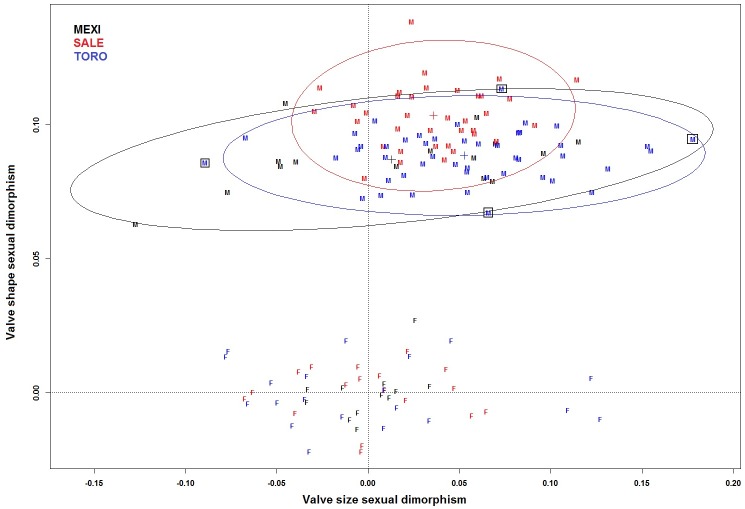
Valve shape by size sexual dimorphism. Valve shape (log[L/H]) and size (log[area]), expressed as deviations from the mean of each species female population. Deviations from the origin in the horizontal and vertical direction represent magnitudes of size and shape dimorphism, respectively. Black boxes indicate four extreme specimens of *C*. *torosa* shown in Fig 4. M = male specimen, F = female specimen.

Male *Cyprideis* valves are significantly larger than females in *C*. *salebrosa* and *C*. *torosa* (SALE, two-sample *t* = -3.45, *df* = 28.87, *p =* 0.002; TORO, two-sample *t* = -3.28, *df* = 31.63, *p =* 0.002, respectively) but not in *C*. *mexicana* (MEXI, two-sample *t* = -0.67, *df* = 16.93, *p =* 0.515). As expected, males are significantly more elongate (higher L:H ratio) than females in all three species (MEXI, two-sample *t* = -21.59, *df* = 24.93, *p <<* 0.001; SALE, two-sample *t* = -33.92, *df* = 38.59, *p <<* 0.001; TORO, two-sample *t* = -29.95, *df* = 29.09, *p <<* 0.001; Fig 3, Table 2).

**Table 2:** Mean (m, in μm for linear dimensions, μm^2^ for area), coefficient of variation (CV) and sample size for the left (L) valve length, height and area, and bilateral dimorphism (L-R), with 95% confidence intervals (CI), in male and female *Cyprideis*. Entries with *p* < 0.05 indicated in bold.

			Valve Length		Valve Height		Valve Length/Height		Area		
Species	Sex		m	CV 95%CI	m	CV 95%CI	m	CV 95%CI	m	CV 95%CI	N
*C*. *mexicana* (MEXI)	F	L mean	909	1.4	522	0.9	1.7	1.2	371380	2	12
		**L-R**	**10.1**	8.4–11.8	**19.4**	18.2–20.6	**-0.1**	-0.1–-0.09	35905	33668–38142	12
	M	L mean	958.2	4.2	497.8	3.5	1.9	1.3	372098	7.5	14
		**L-R**	**10.6**	8.8–12.3	**13.7**	12.5–15	**-0.07**	-0.08–-0.06	28957	26300–31614	14
*C*. *salebrosa* (SALE)	F	L mean	1165	2.0	721.6	2.2	1.6	1.1	658165	4.1	18
		**L-R**	**6**	4.3–7.6	**19.5**	17.9–21.1	**-0.07**	-0.08–-0.07	43025	39358–46692	17
	M	L mean	626.6	1.8	698	1.6	1.8	1.3	683550	3.2	33
		**L-R**	**5.8**	4.8–6.9	**19**	18.1–20	**-0.09**	-0.00–-0.08	44663	42650–46676	27
*C*. *torosa* (TORO)	F	L mean	1012	3.4	784.8	3.2	1.7	1.3	462344	6.6	20
		**L-R**	**14.3**	12.6–16	**15.6**	14.9–16.2	**-0.05**	-0.05–-0.04	39448	37526–41370	20
	M	L mean	1084	2.9	573.8	2.7	1.9	1.0	484555	5.5	47
		**L-R**	**8.8**	7.7–10	**14.2**	13.7–14.7	**-0.07**	-0.07–-0.06	34804	33426–36182	45

We found rather subtle bilateral asymmetry in the soft anatomy, with the left side, overall, having larger values in the reference limbs (Table 3; raw measurements available as S2 Table) and the right side with larger values in the hemipenis (Table 4, S3 Table), with some variation across limbs and species.

**Table 3:** Mean (m, in μm), coefficient of variation (CV) and sample size for left (L) limb of each reference character and directional asymmetry (DA, L-R), with 95% confidence intervals (CI), in male and female *Cyprideis*. Four non-sexually dimorphic characters were selected in female (F) and male (M) *Cyprideis*: 1^st^ and 2^nd^ antenna, mandibula and 3^rd^ walking leg (A1, A2, Md, WL3, respectively). Nomenclature following Table 1; entries with *p* < 0.05 indicated in bold.

			1A			2A			Md			3WL		
Species	Sex		m	CV 95%CI	N	m	CV 95%CI	N	m	CV 95%CI	N	m	CV 95%CI	N
*C*. *mexicana* (MEXI)	F	L mean	116	2.4	11	123	4.3	8	264	1.6	11	140	4.4	2
		**L-R**	-1.3	-3.5–0.9	10	0.9	-0.9–2.5	8	2	-1.2–5.3	10	0.2	-17.4–17.8	2
	M	L mean	121	4	12	135	5.2	10	268	4.6	14	142	4.5	12
		**L-R**	0.4	-2.2–1.4	11	-1	-3.5–1.6	10	**3.5**	1–5.9	13	-2	-3.2–0.7	10
*C*. *salebrosa* (SALE)	F	L mean	153	3.4	15	184	2.8	16	312	2.1	9	186	3.2	16
		**L-R**	0.8	-0.5–2.2	14	0	-2.4–2.3	15	-0.9	-3.4–1.7	3	0.7	-1.1–2.4	16
	M	L mean	156	1.8	29	191	2.7	28	318	2.3	18	187	2.9	31
		**L-R**	0.2	-1–1.5	26	**2**	0.6–3.5	27	**3.7**	0.6–6.9	16	0.1	-1.4–1.6	27
*C*. *torosa* (TORO)	F	L mean	132	3.0	20	159	3.4	20	282	2.6	13	161	3.1	19
		**L-R**	2.3	-1.1–3.5	20	1.4	-0.3–3.1	19	**4.5**	0.9–8.1	10	1.6	0–3.3	19
	M	L mean	134	2.5	45	163	2.4	45	290	2.9	41	168	3.1	43
		**L-R**	**1.1**	0.3–1.8	45	**1.1**	0.2–2.1	44	**2.2**	0.1–4.3	22	3.1	1.9–4.4	41

**Table 4:** Mean (m, in μm for linear dimensions, μm^2^ for area), coefficient of variation (CV) and sample size (N) of the size of male primary sexual trait and directional asymmetry (L-R), with 95% confidence intervals (CI), and directional asymmetry (DA) of the 1WL with 95% confidence intervals in male *Cyprideis*. The length of the Basal capsule distal chitinized support (HemiBCd L) and Copulatory process (HemiTE L), and area of the Terminal extension section (HemiTE A) for the left (L) side are reported. Bilateral dimorphism of the 1WL length (WL1 L; calculated as L-R) and width (WL1 W, calculated by subtracting L (Length curve—length straight line) from R (Length curve—length straight line)) is indicated to best illustrate degree of sexual dimorphism in the secondary sexual character. Abbreviations follow Table 1; entries with *p* < 0.05 indicated in bold.

***Hemipenis***			**HemiBCd L**			**HemiTE L**			**HemiTE A**		
**Species**			**m**	**CV 95%CI**	**N**	**m**	**CV 95%CI**	**N**	**m**	**CV 95%CI**	**N**
*C*. *mexicana* (MEXI)	L mean		417	4.0	10	109	4.0	14	21037	10.6	12
	**L-R**		-3.1	-9.0–2.9	10	0.8	-0.9–2.4	14	-270.4	-1020.8–480.0	11
*C*. *salebrosa* (SALE)	L mean		551	2.9	31	180	3.1	25	83333	6.6	18
	**L-R**		-1.4	-5.9–3.1	30	-**9.2**	-12.9–-5.6	24	38.0	-1250.8–1326.8	18
*C*. *torosa* (TORO)	L mean		491	3.4	47	136	3.5	45	25143	5.3	45
	**L-R**		**-5.4**	-8.0–-2.9	47	-1.8	-3.6–0.0	44	**-1689.9**	-2071.0–-1308.7	44
***Secondary sexual trait***			**1WL W**			**1WL L**					
**Species**	**Sex**		**DA**	**95%CI**	**N**	**DA**	**95%CI**	**N**			
*C*. *mexicana* (MEXI)	F	**L-R**	-	-	0	-	-	0			
	M	**L-R**	**-8.7**	-10.9–-6.3	10	**-5.2**	-7–-3.3	8			
*C*. *salebrosa* (SALE)	F	**L-R**	-0.1	-1.1–0.9	15	0.2	-0.5–0.1	15			
	M	**L-R**	**-12.9**	-13.9–-11.9	31	**-8.4**	-8.9–-7.8	32			
*C*. *torosa* (TORO)	F	**L-R**	0.2	-0.5–0.9	16	-0.1	-0.3–0	16			
	M	**L-R**	**-6.8**	-7.3–-6.3	41	**-10.8**	-11.5–-10.1	41			

**Fig 4:**
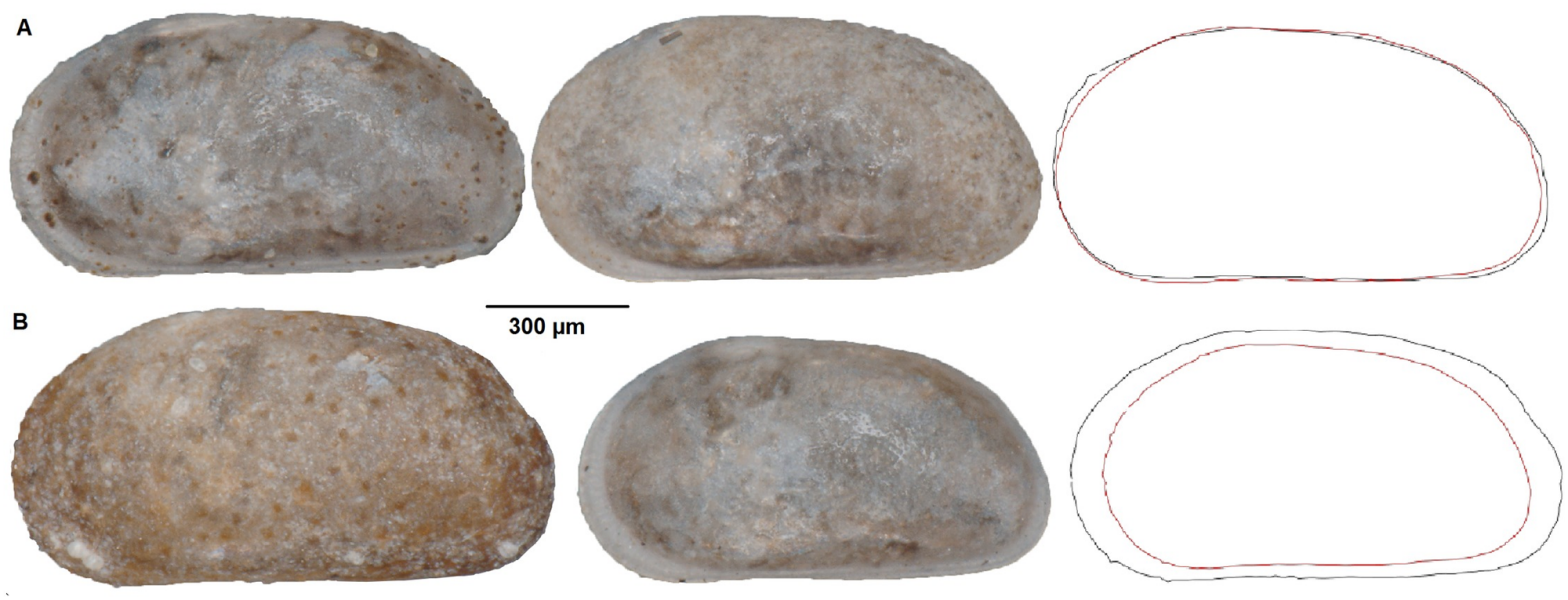
Left valves and outlines representing extremes in size and shape for male *Cyprideis torosa*. (A) From left to right, most elongate male, least elongate male, and their overlain outlines. (B) From left to right, the largest male, the smallest male, and their overlain outlines.

Variation in valve size (area) is moderate within sexes; coefficients of variation (CVs) range from 2 to 7.5 across males and females of the three species (Table 2). CVs for L/H are all quite low (≈ 1, Table 2), indicating that there is rather more variation in size than shape within sexes (Fig 4). CVs suggest low variation in the soft anatomy, with values ranging from 2–3. The highest variation is in the male *C*. *mexicana* measurements as a result of a tail of small males in that sample (Table 3). CVs are only slightly higher in the primary sexual characters (Table 4, S3 Table).

### Allometry

**Fig 5:**
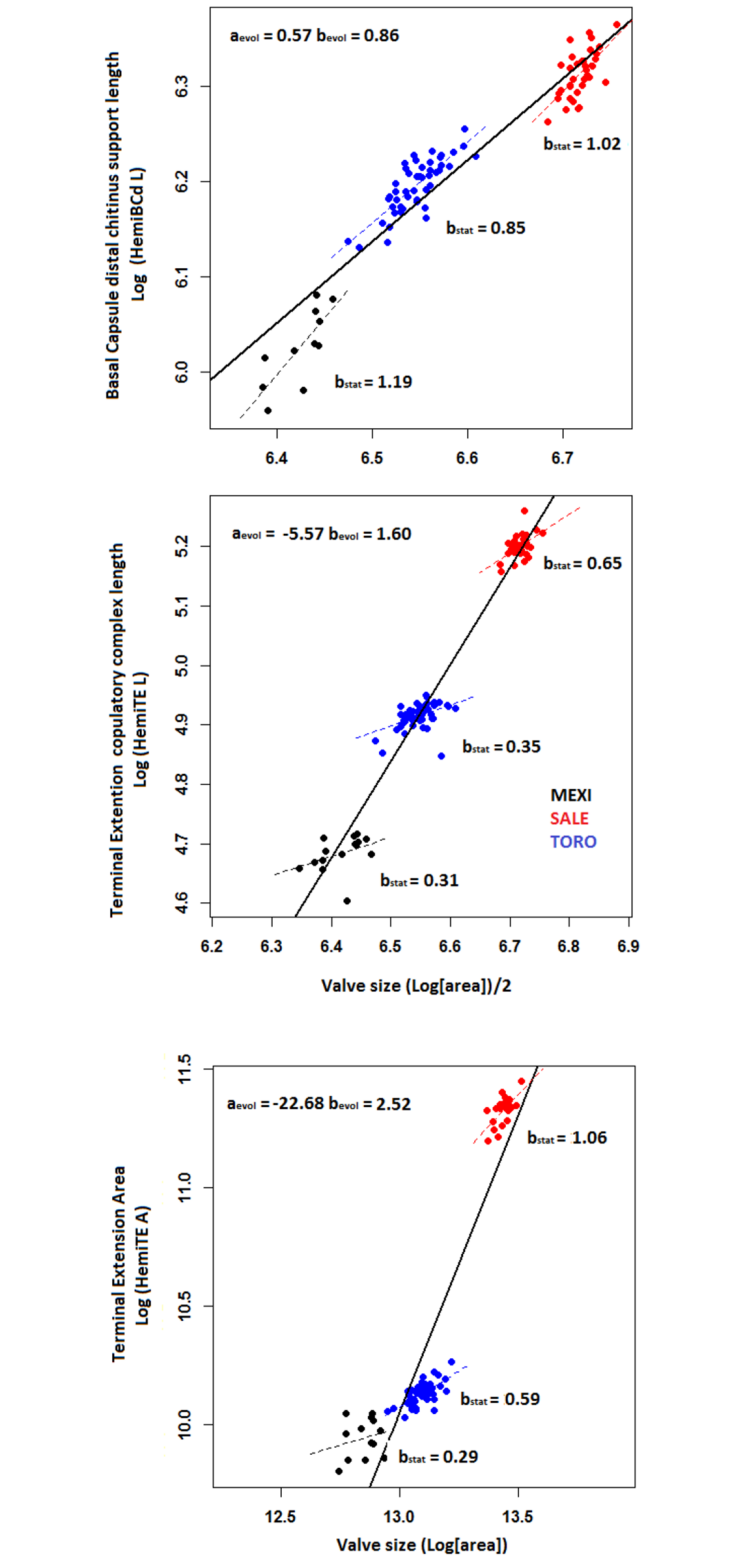
Evolutionary (evol) and static (sta) allometry of the variables representing size in the hemipenis and valve area, used as a proxy for body size. The basal capsule is represented by the Basal Capsule dorsal chitinous support length (HemiBCd L, upper panel); the terminal extension is represented by the Copulatory process length (Hemi TE L, middle panel), and the Terminal extension area (HemiTE A, lower panel). Abbreviations follow Table 1.

Measurements from non-sexual limbs tend to show negative static (within-species) allometries in the species with the highest sample sizes (Table 5), with no trend evident in the species with the lowest sample sizes, *C*. *mexicana*. Among hemipenis characters, the size of the basal capsule is moderately correlated with body size but its allometric relation with respect to valve size varies across all three species (Table 5). Measurements of the terminal extension have weaker correlations with body size, showing low allometric slopes in all three species (Fig 5), except for TE area vs. shell size in *C*. *salebrosa*, which shows a nearly isometric relationship.

**Table 5:** Static allometry of the reference limbs lengths and distinct measurements of the sexual characters using valve area as a proxy for body size. References limbs: 1^st^ and 2^nd^ antenna, mandibula and 3^rd^ walking leg (A1, A2, Md, WL3, respectively); primary sexual traits: Basal Capsule distal chitinous support length, Copulatory complex length and Terminal Extension area (HemiBCd L, HemiTE L, HemiTE A, respectively). Abbreviations follow Table 1; *p*-value: n.s. *p* > 0.05, *p < 0.05, ** *p* < 0.01.

	*C*. *mexicana* (MEXI)			*C*. *salebrosa* (SALE)			*C*. *torosa* (TORO)		
Character	a_intercept_ (95% CI)	b_slope_ (95% CI)	r^2^_adj_	a_intercept_ (95% CI)	b_slope_ (95% CI)	r^2^_adj_	a_intercept_ (95% CI)	b_slope_ (95% CI)	r^2^_adj_
*Reference limbs*									
A1	1.21 (-1.08–3.50)^n.s.^	0.56 (0.07–0.83)**	0.27	-0.22 (-1.90–1.47)^n.s.^	0.78 (0.53–1.03)**	0.42	0.81 (0.03–1.58)*	0.63 (0.51–0.74)**	0.62
A2	-3.01 (-8.18–2.02)^n.s.^	1.23 (0.44–2.03)**	0.29	-1.56 (-3.63–0.52)^n.s.^	1.01 (0.70–1.32)**	0.45	1.00 (0.00–1.98)*	0.63 (0.48–0.78)**	0.51
Md	-1.31 (-2.98–0.37)^n.s.^	1.07 (0.81–1.33)**	0.73	0.13 (-1.60–1.86)^n.s.^	0.84 (0.58–1.09)**	0.47	0.71 (-0.35–1.78)^n.s.^	0.76 (0.59–0.92)**	0.58
3WL	-1.14 (-3.34–1.07)^n.s.^	0.95(0.60–1.29**	0.56	-0.73 (-2.61–1.15)^n.s.^	0.89 (0.61–1.17)**	0.43	0.08 (-1.00–1.16)^n.s.^	0.77 (0.60–0.93)**	0.56
*Primary sexual characters*									
HemiBCd L	-1.62 (-6.32–3.08)^n.s.^	1.19 (0.56–1.92)**	0.56	-0.57 (-3.58–2.45)^n.s.^	1.02 (0.57–1.47)**	0.38	0.63 (-0.59–1.86)^n.s.^	0.85 (0.66–1.04)**	0.64
HemiTE L	2.67 (-0.25–5.58)^n.s.^	0.31 (-0.14–0.77)^n.s.^	0.08	0.81 (-2.07–3.69)^n.s.^	0.65 (0.23–1.08)*	0.23	2.61 (1.25–3.97)**	0.35 (0.14–0.56)**	0.19
HemiTE A	6.20 (-4.93–17.33)^n.s.^	0.29 (-0.57–1.16)^n.s.^	-0.04	-2.94 (-11.19–5.32)^n.s.^	1.06 (0.45–1.68)**	0.38	2.41 (-1.11–4.95)^n.s.^	0.59 (0.40–0.78)**	0.44

Evolutionary allometries (between-species) for the hemipenis are reported in Fig 5, but we do not interpret them in detail as they are based on patterns from only three species of unknown phylogenetic relationship. We do observe that the basal capsule and the terminal extension yield very different evolutionary allometries (Fig 5): the former have negative allometry whereas the latter shows strong positive allometry, mostly driven by the very large terminal extension in *C*. *salebrosa*, the largest of the three species.

Composite softpart size is highly correlated with valve area, both overall (*r* = 0.98, *df* = 146, *p <<* 0.001) and within each individual species (SALE: *r* = 0.82, *df* = 52, *p <<* 0.001; TORO: *r* = 0.88, *df* = 65, *p <<* 0.001; MEXI: *r* = 0.79, *df* = 25, *p <<* 0.001).

### Correlation between sexual traits and valve sexual dimorphism

**Fig 6:**
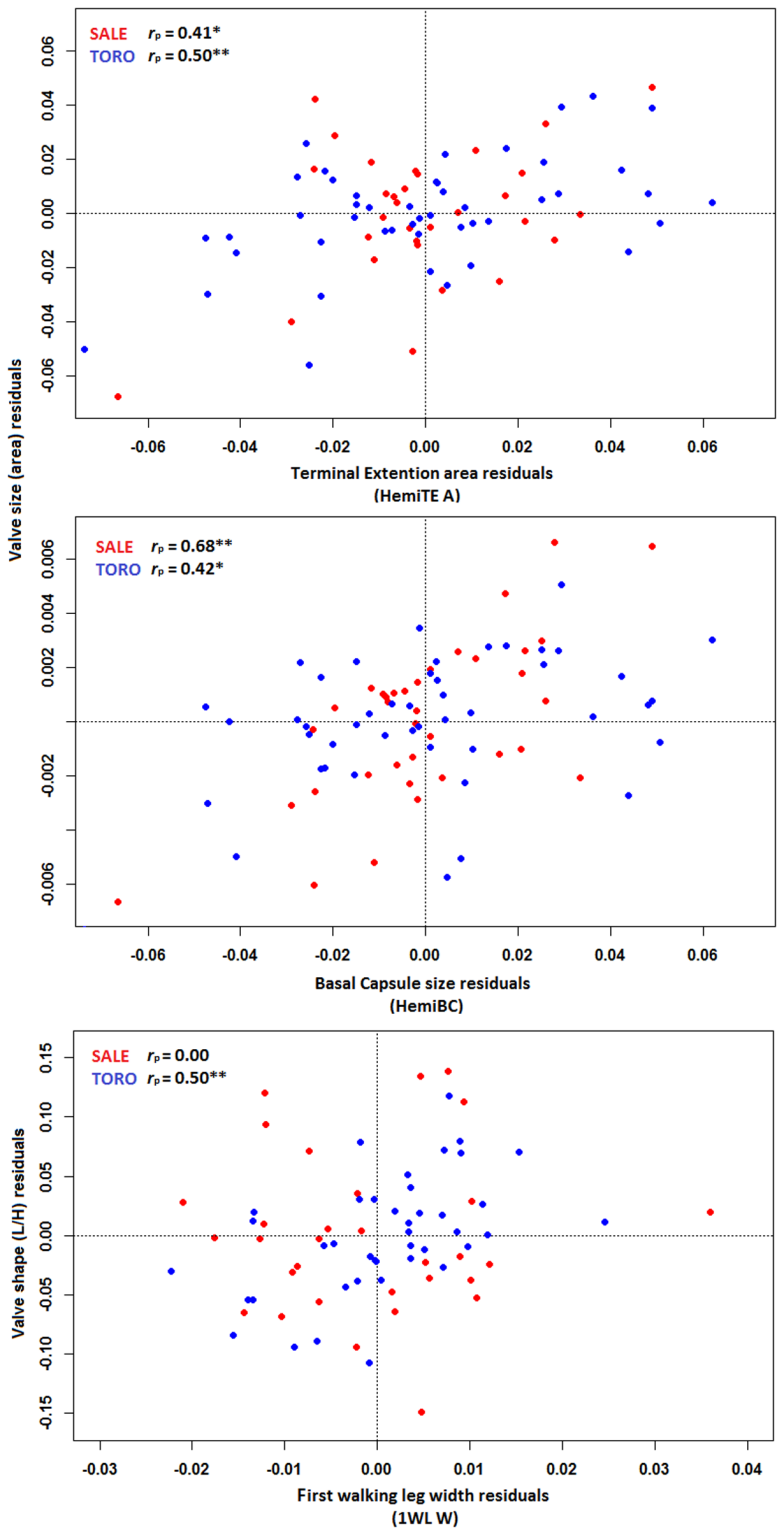
Selected male trait—Valve relationships. Shown are partial correlations between valve size (log[area]) and shape (log[Length/Height]) and male sexual traits, accounting for their shared relationship to softpart size. Residuals are from regressions of each variable on softpart size, as explained in the text. The terminal extension is represented by the Terminal extension area (HemiTE A, upper panel); the basal capsule is represented by the average obtained for the three lengths taken (HemiBC, middle panel); and the first walking leg is represented by the width difference between left and right side (1WL W, lower panel). Abbreviations follow Table 1.

Partial correlations suggest that valve size in males is related to the size of the male copulatory structures, even after accounting for their shared relationship with softpart size (Table 6). The pattern is strongest for the size of the basal capsule and for the area of the terminal extension, and weakest for the length of the intromittent organ (HemiTE L) in *C*. *salebrosa* and *C*. *torosa* (Fig 6a and 6b). On the other hand, there is little indication that relatively large hemipenis dimensions are correlated with valve elongation; except for HemiBC in the small sample (*n* = 15) of *C*. *mexicana*, none of these correlations is significant (Table 6). *Cyprideis mexicana* has a limited number of males, with a cluster of rather small males. The greater noise of this small sample may account for differences between this species and the other two.

**Table 6:** Male trait—Valve relationships. Shown are partial correlations between valve size (log[area]) and shape (log[Length/Height]) and male sexual traits, accounting for their shared relationship to softpart size. The primary sexual trait is represented by the Basal Capsule size, and the Copulatory complex length and Terminal extension area (HemiBC, HemiTE L, HemiTE A, respectively) and the first walking leg is represented by the width and curvature difference between left and right side (1WL W and 1WL L, respectably). Abbreviations follow Table 1; entries with *p* < 0.05 indicated in bold.

	Valve trait	
Male trait	Valve size (log[area])	Valve shape (log[L/H])
HemiBC	**TORO: *r***_***p***_ **= 0.42, *df* = 45, *P* = 0.003** **SALE: *r***_***p***_ **= 0.68, *df* = 34, *P* = 8.4e-6** MEXI: *r*_*p*_ = 0.34, *df* = 13, *P* = 0.228	TORO: *r*_*p*_ = 0.10, *df* = 45, *P* = 0.51 SALE: *r*_*p*_ = -0.06, *df* = 34, *P* = 0.74 **MEXI: *r***_***p***_ **= 0.57, *df* = 13, *P* = 0.03**
HemiTEA	**TORO: *r***_***p***_ **= 0.50, *df* = 45, *P* = 0.0004** **SALE: *r***_***p***_ **= 0.41, *df* = 29, *P* = 0.024** MEXI: *r*_*p*_ = -0.45, *df* = 13, *P* = 0.11	TORO: *r*_*p*_ = -0.08, *df* = 45, *P* = 0.61 SALE: *r*_*p*_ = -0.13, *df* = 29, *P* = 0.50 MEXI: *r*_*p*_ = -0.00, *df* = 13, *P* = 0.99
HemiTE L	TORO: *r*_*p*_ = 0.08, *df* = 45, *P* = 0.59 **SALE: *r***_***p***_ **= 0.43, *df* = 29, *P* = 0.02** MEXI: *r*_*p*_ = -0.22, *df* = 13, *P* = 0.45	TORO: *r*_*p*_ = 0.02, *df* = 45, *P* = 0.88 SALE: *r*_*p*_ = -0.11, *df* = 29, *P* = 0.55 MEXI: *r*_*p*_ = 0.04, *df* = 13, *P* = 0.90
WL1 W	TORO: *r*_*p*_ = -0.02, df = 39, *P* = 0.86 SALE: *r*_*p*_ = 0.11, *df* = 29, *P* = 0.55 MEXI: NA, *df* = 8	**TORO: *r***_***p***_ **= 0.50, *df* = 39, *P* = 0.0009** SALE: *r*_*p*_ = -0.00, *df* = 29, *P* = 0.99 MEXI: NA, *df* = 8
WL1 L	TORO: *r*_*p*_ = 0.11, *df* = 39, *P* = 0.51 SALE: *r*_*p*_ = -0.20, *df* = 30, *P* = 0.28 MEXI: NA, *df* = 6	**TORO: *r***_***p***_ **= 0.42, df = 39, P = 0.006** SALE: *r*_*p*_ = 0.12, *df* = 30, *P* = 0.52 MEXI: NA, *df* = 6

The degree of modification of the first walking leg into a clasping organ is not associated with valve size, but it is associated with valve shape in *C*. *torosa*: males with more strongly modified first walking legs have relatively more elongate valves (Table 6, Fig 6c).

## Discussion

In this study, we apportioned sexual dimorphism of the valves into two dimensions: size and shape dimorphism. These two components may evolve independently (e.g., [37]), and indeed yield different correlations with anatomical traits. Our most noteworthy results concern the moderate but significant correlations between valve sexual size dimorphism and the size of distinct structural parts of the hemipenis. In contrast, valve shape dimorphism was not correlated with size measurements of the hemipenis parts in the two species with high sample sizes. Thus larger males but not more elongate ones, have larger hemipenis components, even after accounting for the tight correlation between shell and body size. This relationship is most consistent for the basal capsule, the muscular, sperm-pumping component of the male genitalia, suggesting that sexual size dimorphism may be related to the size or quantity of sperm, or to the efficiency with which sperm may be pumped.

At 200–215 μm in length [18], sperm in *C*. *torosa* are moderate in size if compared with the giant sperm of cypridoid ostracodes [19], but they are still quite large relative to body size (~20%). We were not able to measure sperm size in the present study, but a significant positive correlation was detected among sperm length, sperm pump size, and valve length within candonid ostracodes (though note that the sperm pump in candonids is a structure separate from the copulatory apparatus, and not homologous with the basal capsule) [19]. Although not consistent across all Ostracoda, this finding does suggest that the size of the sperm pump may be related to sperm size in at least some groups. In other taxa such as *Drosophila*, longer sperm can have higher fertilization success compared to smaller sperm [25, 38, 39].

Like the basal capsule, the area of the terminal extension of the hemipenis also shows significant partial correlations with male valve size for *C*. *torosa* and *C*. *salebrosa*; the same finding for the length of the intromittent organ on the terminal extension holds for *C*. *salebrosa* only. As these are the parts of the male genitalia in contact with females during mating, these structures are more likely to be influenced by sexual selection factors related to mate recognition or sexual conflict with females. Martens [40] proposed that variation in sexual traits across lineages in *Limnocythere*, particularly parts of the copulatory complex that show clear species-specific shape, plays a key role in species recognition, supporting a scenario as envisioned in our study system. The different patterns of static allometry between the basal capsule and the terminal extension suggests that these structures may indeed experience distinct evolutionary dynamics, as does the observation that the basal capsule is conserved across members of the genus whereas the terminal extension is highly divergent in size and shape [30]. Similar dynamics of genital evolution are known in *Drosophila* (e.g., [41]) and in the beetle *Onthophagus* (e.g., [42, 43]). Further investigation of the shape of the copulatory process in *Cyprideis*, and not just its size, has the potential to offer more insight. It is challenging to assess sexual conflict in ostracodes because there have been few attempts to study female genitalia and consequently the coevolution of male and female genitalia [20]. Unlike the male genitalia, female reproductive structures are membranous (see [32]) and their size and shape are not easily characterized.

Although we have shown here that the degree of valve sexual size dimorphism in *Cyprideis* is partly related to the size of the male genitalia, genital size is only one of many factors that may influence male body size. In addition to ecological and environmental factors (for *C*. *torosa*, see [44, 45]), body size may also be important in precopulatory sexual selection. In male-contest situations in other taxa, larger males generally have an advantage [7], and thus adult male body size may reflect sexual selection that is both precopulatory (through male-male contests) and post-copulatory (via possible differences in sperm as discussed above) (see also [16]). Indeed, investment in sexual traits has also been hypothesized to reflect male condition, with the highest quality individuals displaying the largest and most elaborate character [7]. However, in our system, it is difficult to choose between these alternative mechanisms as virtually no studies have been attempted on this topic in ostracodes.

In cytheroids, including the genus *Cyprideis* [28], species differences in reproductive structures were assumed to affect valve sexual dimorphism, namely male valve elongation as a consequence of the need to accommodate their large copulatory apparatus. However, the shape of the male valves does not seem to be associated with the aspects of genital size that we measured. The sole significant correlation, the size of the basal capsule in *C*. *mexicana*, is based on a small sample (*n* = 15), and we found little evidence that, in general, more elongate males have disproportionately large genitalia relative to the size of other soft-parts. It should be noted, though, that there is rather little intraspecific variation in male valve shape in all three species. Correlations between valve shape and male reproductive structures might be easier to detect using larger, between-species differences. In a small sample from two sister species of the ostracode genus *Loxoconcha*, Kamiya [46] found that the relatively more elongate species also had a proportionately large hemipenis. Similarly, Danielopol [47] found that hemipenis shape was related to carapace shape in interstitial candonine ostracodes. We are unable to pursue this angle in the present study because it turned out that all three species we examined had very similar magnitudes of shape dimorphism (Fig 3). Broader sampling within the genus *Cyprideis* may help to resolve the relationship between shape dimorphism and male investment in reproductive structures.

In *C*. *torosa*, males with more dimorphic secondary sexual trait (first walking leg) tend to have more elongate valves. Because the right first walking leg is hypothesized to be a clasping structure, this may suggest that valve shape may affect the mechanics of male-female copulation. Little information on species-specific mating position is available in ostracodes [16, 27] although species-specific differences may occur [16]. Within the genus *Cyprideis* no information is available on courting and mating position and species-specific differences are unknown. However, and while the specific mechanisms leading to a chosen mating position are difficult to ascertain [26], it is conceivable that both valve shape and grasping limbs may influence this process [16, 26]. Alternatively, other processes related (or not) to male investment in reproduction may influence valve shape (e.g., [48]).

Our attempts to relate valve morphology to male sexual structures may have been complicated by differences between the sexes unrelated to sexual selection. In *Cyprideis*, females keep their eggs and first instar juveniles in brooding areas that result in more laterally inflated valves in females, but this apparently does not affect the shape in lateral view [28]. Also, female body size is correlated with fecundity in insects and other crustaceans, and has been hypothesized to do so in ostracodes as well (e.g., [49]). Natural selection on female fecundity might influence female body size, and therefore sexual size dimorphism as well.

We confirm here a strong correlation between the size of soft-parts and the overall size of the mineralized valves in three species of *Cyprideis*. This finding indicates that valve size is a useful proxy for the size of non-mineralized structures of ostracodes, and presumably, body mass. We also note that we detected subtle directional asymmetry in several of the limbs, with appendages on the left side generally being larger than those on the right. To our knowledge, this pattern has not previously been reported in ostracodes. Evidence for asymmetry is strongest in *C*. *torosa*, the species with the highest sample sizes, and it is seen more consistently in the mandibula compared to the first and second antennae. Estimates of directional asymmetry are most variable when sample sizes are low, but then stabilize with increasing statistical power to values with left-side structures being 0.5–1.0% larger than corresponding structures on the right (S1 Fig). The direction of this asymmetry appears to be reversed in the hemipenis; the three statistically significant asymmetries found for this structure all indicate that the right side is larger than the left side.

## Conclusions

To our knowledge, our study is the first to examine the correlation between investment in primary and secondary sexual traits and valve dimorphism within ostracode species. Our results suggest a complex interaction between hard and soft anatomy, with a direct, moderate correlation between sexual dimorphism of the valve and sexual traits. These results substantiate previous assumptions that valve dimorphism can be used as a proxy for investment in sexual traits. However, it is size dimorphism, rather than the commonly assumed shape dimorphism, that can be linked directly to investment in male sexual traits.

The superfamily Cytheroidea represents a large portion of ostracode diversity, especially in the marine realm. Because of their strongly calcified valves, cytheroids have an extremely rich fossil record that extends back to the Ordovician [50, 51]. Sexual dimorphism of the valves depends on the internal soft anatomy and underlying biology, and thus the fossil record of these structures represents a valuable archive of character evolution. These anatomical characters may evolve in response to extrinsic, environmental factors, to distinct reproductive demands of each sex, and to sexual selection, subject to physiological constraints. Yet sexual dimorphism of the shell, although widely recognized, has largely been neglected in its potential to explore sexual selection through deep time.

## Supporting information

S1 Fig Directional asymmetry estimates with respect to sample size. Asymmetry was computed on a percent scale, 100*[L-R]/L. Limbs are plotted according to their abbreviations in Table 3; colors indicate species (black = *C*. *mexicana*, red = *C*. *salebrosa*, green = *C*. *torosa*) and fonts indicate sex (bold = female, italics = male).
(TIFF)

S1 Table Sample information and number of individuals (N) used in morphometric analyses. *All samples within species were pooled for analysis. Latitude and longitudes are approximate for *C*. *mexicana*.
(DOCX)

S2 Table Raw measurements from valves, sexual and non-sexual limbs. (XLSX)

S3 Table Mean (m, in μm), directional asymmetry (L-R), coefficient of variation (CV) and sample size (N) of the remaining variables analyzed representing size in the hemipenis basal capsule section (HemiBC 1-2L, Hemi 3–4 L). (DOCX)

S1 File Video of *Cyprideis torosa* in laboratory. (ZIP)
