# Supplementary figures and images for: Correlation between investment in sexual traits and valve sexual dimorphism in Cyprideis species (Ostracoda)
Source: PLoS One. 2017 Jul 5;12(7):e0177791. doi: 10.1371/journal.pone.0177791 (PMC5497955; doi:10.1371/journal.pone.0177791)

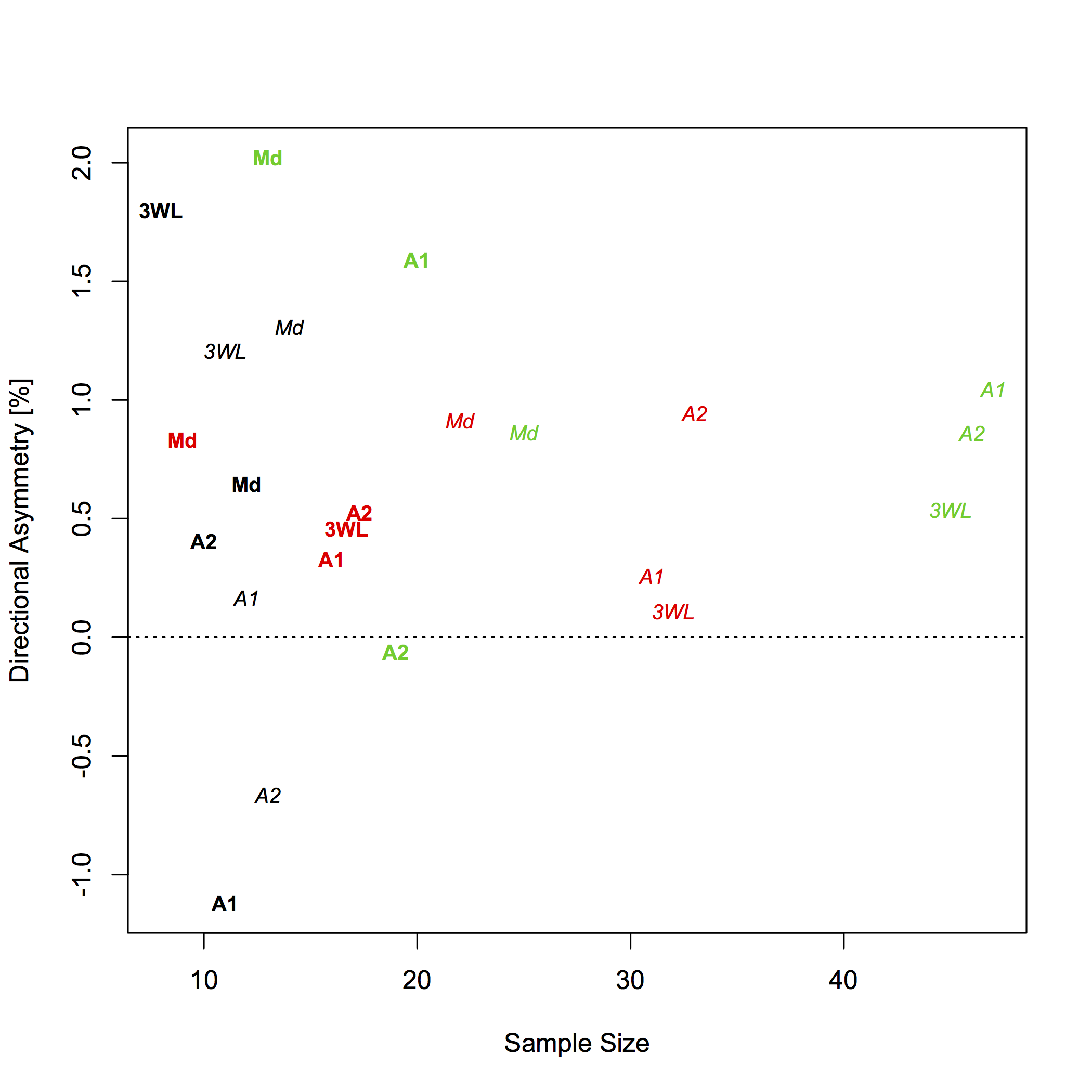

Supplement: S1 Fig — Asymmetry was computed on a percent scale, 100*[L-R]/L. Limbs are plotted according to their abbreviations in Table 3; colors indicate species (black = C. mexicana, red = C. salebrosa, green = C. torosa) and fonts indicate sex (bold = female, italics = male). (TIFF) [file pone.0177791.s001.tiff]
