# Supplementary material for: Correlation between investment in sexual traits and valve sexual dimorphism in Cyprideis species (Ostracoda)
Source: PLoS One. 2017 Jul 5;12(7):e0177791. doi: 10.1371/journal.pone.0177791 (PMC5497955; doi:10.1371/journal.pone.0177791)
Supplement: S1 Table — *All samples within species were pooled for analysis. Latitude and longitudes are approximate for C. mexicana. (DOCX) [file pone.0177791.s002.docx]

| **Species** | **No. samples** | **USNM** | **Location** | **Coordinates** | **Sampling year** | **N male** | **N female** |
| --- | --- | --- | --- | --- | --- | --- | --- |
| *C. mexicana* | 2***** | 128369 | Copano Bay, Texas, USA | 28.12 N, 97.10 W | 1960 | - | 12 |
|  | 2* | 128389 | Redfish Bay,  Texas, USA | 27.87 N, 97.12 W | 1960 | 5 | - |
|  | 1 | 128367 | Laguna Madre, and Copano and Redfish Bay,  Texas, USA |  | 1960 | 10 | - |
| *C. salebrosa* | 3* | 1453056 | Chesapeake Bay, Maryland, USA | 38.89936 N, 76.53967 W | 2013 | 38 | 18 |
| *C. torosa* | 1 | 1453057 | Pegwell Bay, Kent, UK | 51.1921 N, 1.2145 E | 2015 | 47 | 20 |
