# Supplementary material for: Correlation between investment in sexual traits and valve sexual dimorphism in Cyprideis species (Ostracoda)
Source: PLoS One. 2017 Jul 5;12(7):e0177791. doi: 10.1371/journal.pone.0177791 (PMC5497955; doi:10.1371/journal.pone.0177791)
Supplement: S3 Table — (DOCX) [file pone.0177791.s004.docx]

| ***Hemipenis*** | | **HemiBC12 L** | | | **HemiBC34 L** | | |
| --- | --- | --- | --- | --- | --- | --- | --- |
| **species** |  | **m** | **CV**  **95%CI** | **N** | **m** | **CV**  **95%CI** | **N** |
| *C. mexicana* (MEXI) | L mean | 181.0 | 8.3 | 13 | 209.0 | 6.5 | 11 |
|  | **L-R** | -0.4 | -8.6 ̶ 7.8 | 13 | -1.1 | -6.3 ̶ 4.1 | 11 |
| *C. salebrosa* (SALE) | L mean | 248.0 | 2.9 | 34 | 303.0 | 3.5 | 33 |
|  | **L-R** | -0.3 | -3.6 ̶ 3.0 | 34 | -3.7 | -7.3 ̶ -0.2 | 33 |
| *C. torosa* (TORO) | L mean | 197.0 | 3.4 | 46 | 273.0 | 4.0 | 47 |
|  | **L-R** | **-3.1** | -5.3 ̶ -0.9 | 46 | **-4.7** | -6.7 ̶ -2.7 | 47 |
